# Quantitative whole-heart three-dimensional magnetic resonance myocardial perfusion imaging in systole and diastole at 3.0T

**DOI:** 10.1186/1532-429X-15-S1-P206

**Published:** 2013-01-30

**Authors:** Manish Motwani, Ananth Kidambi, Timothy Fairbairn, Akhlaque Uddin, Sebastian Kozerke, Steven Sourbron, John P Greenwood, Sven Plein

**Affiliations:** 1 MCRC & LIGHT, University of Leeds, Leeds, UK; 2 Institute for Biomedical Engineering, University and ETH Zurich, Zurich, Switzerland; 3 Medical Physics, University of Leeds, Leeds, UK

## Background

Two-dimensional (2D) perfusion-CMR has been shown to have greater diagnostic accuracy than single-photon emission computed tomography but remains limited by a lack of complete myocardial coverage. Three-dimensional (3D) whole-heart myocardial perfusion CMR addresses this limitation and has recently been shown to be clinically feasible. However, the feasibility and potential clinical utility of *quantitative* 3D perfusion measurements, as already shown with 2D-perfusion-CMR and positron emission tomography, has yet to be evaluated. The purpose of this study was to establish the feasibility of quantitative 3D-perfusion-CMR to detect coronary artery disease (CAD). Additionally, as 3D-perfusion-CMR offers the opportunity to select the phase of acquisition, a secondary objective was to determine differences between systolic and diastolic estimates of myocardial blood flow (MBF).

## Methods

**Figure 1:**
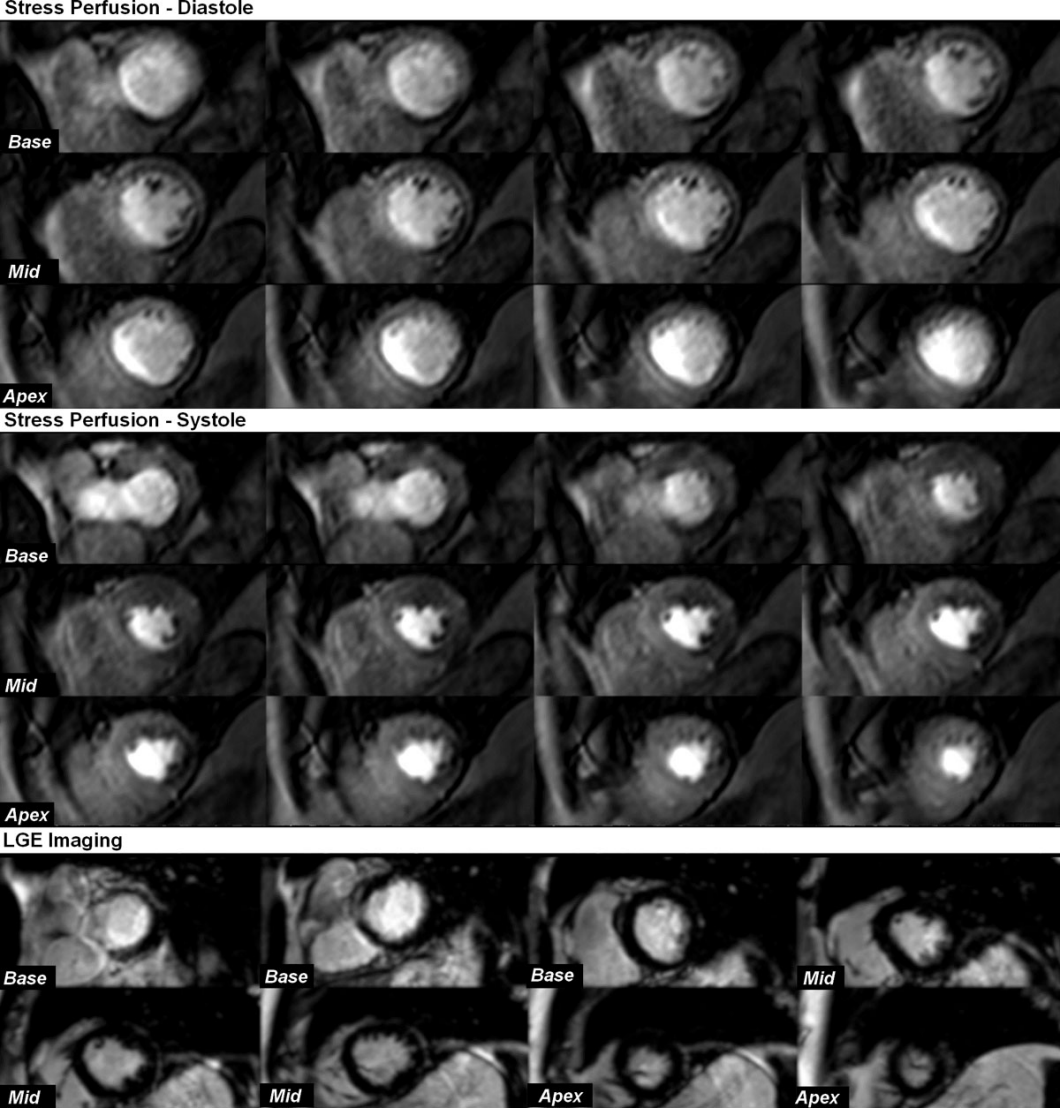
This example shows 3D-perfusion-CMR in a 75-year-old man presenting with angina. Stress-induced perfusion defects are seen infero-laterally from base to apex and antero-laterally from mid-ventricle to apex in both diastole and systole. However, perfusion defects are difficult to discern from dark-rim artifact in diastole and are more clearly delineated with systolic acquisition. Late-gadolinium enhancement imaging did not reveal any previous myocardial infarction. X-ray coronary angiography revealed 80% stenosis of a large diagonal branch and significant proximal disease in a large dominant left circumflex artery.

35 patients underwent 3D-perfusion-CMR (Philips 3T Achieva TX) with data acquired at both end-systole and mid-diastole (Fig 1). Systolic and diastolic perfusion images were analyzed in separate reporting sessions in random order. Image quality (0=non-diagnostic, 1=poor, 2=adequate and 3=excellent) and the occurrence of artifact related to respiratory-motion, k-t reconstruction or dark-rim artifact (0=none, 1=mild, 2=moderate and 3=severe) were scored. MBF and myocardial perfusion reserve (MPR) were estimated on a per patient and per territory basis by Fermi function deconvolution. CAD was defined as luminal stenosis ≥70% on quantitative coronary angiography.

## Results

**Figure 2:**
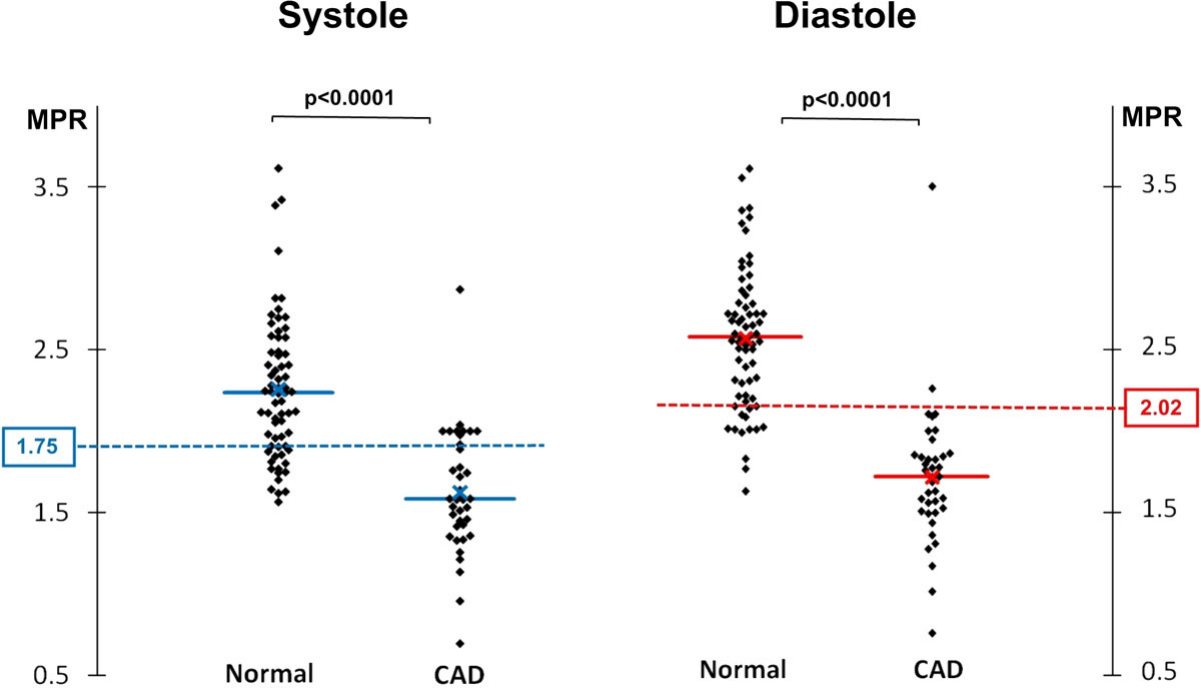
These charts show the individual myocardial perfusion reserve (MPR) values from normal and significantly diseased perfusion territories with both systolic and diastolic acquisition (x=mean value, solid line = median value). The optimal cut-off MPR values determined by receiver-operating characteristic (ROC) analysis are also plotted (dashed lines, 1.75 for systole and 2.02 for diastole). ROC analysis found MPR had a high diagnostic accuracy for the detection of CAD, in both systole and diastole (area under curve: 0.92 vs. 0.94 respectively; p=0.41).

38 coronary territories had significant CAD. MPR had a high diagnostic accuracy for the detection of CAD, in both systole and diastole (area under curve: 0.92 vs. 0.94; p=0.41) (Fig 2). At rest, systolic and diastolic MBF estimates were similar - in both normal and diseased territories (no CAD: 1.24 ± 0.15 vs. 1.25 ± 0.15ml/g/min, p=0.27; CAD: 1.24 ± 0.15 vs. 1.26 ± 0.14ml/g/min, p=0.20). At stress, diastolic MBF estimates were significantly greater than systolic estimates (no CAD: 3.21 ± 0.50 vs. 2.75 ± 0.42ml/g/min, p<0.0001; CAD: 2.13 ± 0.45 vs. 1.98 ± 0.41ml/g/min, p<0.0001). The diastolic/systolic stress MBF ratio was significantly reduced in territories with CAD (CAD: 1.08 ± 0.06 vs. no CAD: 1.17 ± 0.11; p<0.0001). Systolic acquisition had a higher overall image quality score (median: 3 vs. 2, p=0.002) and was less prone to artifact than diastolic acquisition (median artifact score: 0 vs. 1; p<0.0001). In particular, there was a greater frequency of dark-rim artifact in diastole compared to systole (19 vs. 9 patients).

## Conclusions

We have shown that quantitative 3D-perfusion-CMR is feasible and can be used to detect CAD with high diagnostic accuracy. Image quality and less artifact, make systole the preferred phase for acquisition in 3D-perfusion-CMR. Finally, there were significant differences in systolic and diastolic MBF estimates and therefore the phase of acquisition should always be stated in future quantitative studies.

## Funding

JPG and SP receive an educational research grant from Philips Healthcare. SP is funded by a BHF fellowship (FS/1062/28409).

